# Differences in sleep EEG coherence and spindle metrics in toddlers with and without receptive/expressive language delay: a prospective observational study

**DOI:** 10.1186/s11689-024-09586-1

**Published:** 2025-02-22

**Authors:** Xinyi Hong, Cristan Farmer, Nataliia Kozhemiako, Gregory L. Holmes, Lauren Thompson, Stacy Manwaring, Audrey Thurm, Ashura Buckley

**Affiliations:** 1 National Institute of Mental Health, National Institutes of Health, 10 Center Dr, Bethesda, MD 20814 USA; 2 Brigham and Women’s Hospital, Harvard Medical School, 75 Francis St, Boston, MA 02115 USA; 3 Department of Neurological Sciences, University of Vermont, 111 Colchester Ave, Burlington, VT 05401 USA; 4 Department of Speech and Hearing Sciences, Elson S. Floyd College of Medicine, Washington State University, 412 E Spokane Falls Blvd, Spokane, WA 99202 USA; 5 Department of Communication Sciences and Disorders, University of Utah, 390 1530 E #1201, Salt Lake City, UT 84112 USA

**Keywords:** Sleep architecture, Brain development, Cognitive function, Neurophysiology, Diagnostic markers, Language delay

## Abstract

**Background:**

Changes in brain connectivity during development are thought to reflect organizational and maturational processes that correspond to skill acquisition in domains like motor, language, and cognition. This theory is supported by findings in typically developing children as well as observations of abnormal connectivity among children with neurodevelopmental differences. However, few coherence studies have capitalized on the potential of sleep electroencephalogram (EEG) to examine the developing brain, especially among very young children for whom formal neurodevelopmental diagnosis is not yet possible. Sleep microarchitecture in young children may offer key insights into neurophysiological abnormalities associated with neurodevelopmental trajectories and potentially aid in early detection and intervention. In this study, we explored sleep EEG coherence and sleep spindles in typically developing toddlers and toddlers at increased risk of later neurodevelopmental diagnoses.

**Methods:**

We investigated EEG coherence and sleep spindles in 16 toddlers with receptive and expressive language delay (LangD) and 39 typically developing (TD) toddlers. Participants were aged 12–22 months at baseline, and 34 (LangD, n=11; TD, n=23) participants were evaluated again at 36 months of age.

**Results:**

Average EEG coherence was stronger in the LangD group than the TD group, with differences most prominent during slow-wave sleep. Some age-related increases in coherence were observed, but these did not differ between groups. Sleep spindle density, duration, and frequency changed between baseline and follow-up for both groups, with the LangD group demonstrating a smaller magnitude of change than the TD group. The direction of change was frequency band-dependent for both groups.

**Conclusions:**

These findings indicate that atypical sleep EEG connectivity and sleep spindle development can be detected in toddlers at risk of later neurodevelopmental diagnoses.

**Trial registration:**

https://clinicaltrials.gov/study/NCT01339767; Registration date: 4/20/2011.

**Supplementary Information:**

The online version contains supplementary material available at 10.1186/s11689-024-09586-1.

## Background

Electroencephalogram (EEG) is an important tool for studying the developing brain, offering temporal resolution and insight into functional relationships that other methods like magnetic resonance imaging cannot provide. Functional brain connectivity, or the degree of temporal correlation between two spatially distant neuronal populations, informs our understanding of interactions between regions or networks [1]. EEG coherence is a metric of such connectivity and is expressed as the temporal relationship between the phase shift and amplitude ratio of two electrodes [2].

EEG research has demonstrated a relationship between network topology and cortical maturation in newborns [3] and that resting state functional connectivity increases during infancy [4, 5] and early childhood [6]. These changes are thought to reflect organizational and maturational processes that correspond to skill acquisition in developmental domains like motor [7], cognition [8, 9], and language [10]. By the same token, abnormal coherence has been observed among children with a range of neurodevelopmental differences such as learning disorders [11], speech disorders [12], attention deficit hyperactivity disorder [13], and autism spectrum disorder (ASD) [14, 15]. However, the nature of those EEG coherence abnormalities is varied. Perhaps depending on EEG methodology (e.g., calculation of coherence, brain region, frequency band used) or composition of the sample (e.g., age, comorbidities), children with neurodevelopmental differences have been documented to have both more and less coherence than their typically developing counterparts [13, 14].

Importantly, however, most research on the relationship of EEG coherence to neurodevelopmental differences has been conducted within a (waking) resting state paradigm and in older children/adolescents. This paucity of sleep EEG coherence data in early childhood holds both scientific and logistical promise. Sleep is crucial for the development of physiological and cognitive processes in early childhood. Early on, it plays a key role in building synapses and refining circuits central to motor, language, and social development, and later it becomes essential to learning and memory [16]. In contrast with resting state or task-based paradigms, sleep EEG affords the opportunity to capture neural activity in a way that is unaffected by signal noise from external waking stimuli. This allows for a more sensitive and robust way to image early circuit development, particularly in very young children for whom it may otherwise be difficult. Nevertheless, very few of the many neuroimaging studies investigating the functional and structural underpinnings of neurodevelopmental disorders have capitalized on the potential of sleep EEG to examine the developing brain [15].

Other aspects of sleep EEG may also be related to neurodevelopmental differences. Sleep spindles are a hallmark of NREM sleep presenting as brief bursts of waxing-and-waning oscillations between 10 to 16 Hz [17]. Sleep spindles are believed to have functional roles in information processing, memory consolidation, and neuroplasticity during development [18–20]. Because spindle properties are known to change throughout the lifespan, they can provide insight into the neuro-maturational trajectories of individuals with neurodevelopmental disorders [21]. There is preliminary evidence that sleep spindles are related to developmental impairments in older children and adolescents, such as intellectual disability [22] and ASD [23–25]. However, due to the paucity of longitudinal data in early childhood, differences in developmental trajectories of sleep spindles in children with and without neurodevelopmental disorders are currently unknown. Furthermore, the relationship between other spindle features such as frequency and chirp (intra-spindle frequency change) to developmental trajectory is largely unexplored. These oscillations reflect thalamo-cortical activity, and their internal frequency variation may hold relevant information for understanding neurophysiology and exploring brain pathology in neurodevelopmental disorders [26]. One recent cross-sectional investigation documented more negative chirp in typically developing children as compared to children with ASD (ages 1 – 8 years) [27], suggesting that this may be a fruitful area of investigation. By examining spindle density, frequency, and chirp in combination, this study aims to provide a more comprehensive assessment of the sleep spindle features in early childhood, facilitating the identification of potential markers for language and cognitive impairments, and contributing to our understanding of the neural substrates underlying these conditions.

In summary, the examination of sleep micro-architecture during the early childhood period could offer key insights into neurophysiological abnormalities that are associated with atypical neurodevelopmental trajectories and could aid in early detection and intervention. However, the existing literature is limited – few EEG studies of coherence and other features during sleep are available for the early childhood period, with or without neurodevelopmental differences. With this study, we aimed to address this paucity of data using a longitudinal natural history study of toddlers with and without receptive and expressive language delay. Language delays are one of the earliest observable manifestations of atypical development in children for whom young age precludes neurodevelopmental diagnoses. In the absence of genetic, congenital, or perinatal cause for concern, language delays are one of the earliest behavioral signs of neurodevelopmental disorder brought to the attention of providers [28]. While language delays in most children resolve without intervention, children with delays in both receptive and expressive language at age 2 years are likelier to experience poorer outcomes [29]. Thus, this subpopulation of toddlers with both receptive and expressive language delay is enriched for later neurodevelopmental disability and therefore presents an opportunity to explore correlates of neurodevelopmental disability before such diagnoses are assignable.

In the current study, we conducted an exploratory analysis of sleep EEG coherence and spindle characteristics among young children with and without receptive and expressive language delay. Given the extant literature, we hypothesized that toddlers with receptive/expressive language delay would exhibit stronger EEG coherence compared to the typically developing toddlers, and that these differences would be most pronounced during slow wave sleep, in comparison to rapid eye movement sleep stage and waking (resting) EEG. Additionally, we predicted that toddlers with language delay would demonstrate lower levels and more stability in sleep spindle density, frequency, and chirp compared to typically developing toddlers.

## Methods

### Participants

Participants were drawn from a natural history protocol conducted at the NIH Clinical Center in Bethesda, Maryland, USA (protocol number 11-M-0144; NCT01339767). The study was approved by the NIH Institutional Review Board. Consent from the parent or guardian of each child was obtained prior to participation. Inclusion criteria were being between the age of 9 of 21 months, born full-term, no significant medical or motor impairment deemed responsible for the delays (or any known genetic disorder for the typically developing group), and English reported as the primary language spoken in the home. Additionally, at the initial evaluation, eligibility for the language delay (LangD) group included receptive and expressive language T-scores ≥2 standard deviations below average on the Mullen Scales of Early Learning [30] (MSEL), and eligibility for the typically developing (TD) group included T-scores within 1.5 standard deviations of average on all domains of the MSEL.

A total of 62 (LangD, n = 19; TD, n = 43) toddlers enrolled in the natural history study. Participants were included in the current analysis if they had at least one analyzable sleep study, resulting in n=55 toddlers (LangD, n = 16; TD, n = 39) aged 12 to 22 months at their first visit (mean age 17.4±2.8 months) (Table 1). The sample was majority male (n = 34, 62%) and white (n = 36, 65%). Seven (44%) of the LangD participants received a research ASD diagnosis (based on clinical judgment of DSM-5 criteria informed by research-reliable administrations of the Autism Diagnostic Interview and Autism Diagnostic Observation Schedule) during their study participation, but no other formal diagnoses were systematically evaluated. This small sample size precluded investigation of relationships to eventual ASD diagnosis.

**Table 1.** Sample characteristics

	**Typically Developing (TD)**		**Language Delayed (LangD)**	
	Baseline	Follow-up	Baseline	Follow-up
Total N	39	23	17	11
Male sex, n(%)	24 (62%)	15 (65%)	11 (65%)	7 (64%)
Age in years, mean (SD)	1.43 (0.25)	3.10 (0.11)	1.65 (0.11)	3.19 (0.28)
Age range in years	1.05 – 1.78	2.91 – 3.34	1.48 – 1.85	2.82 – 3.82
Ethnicity, n(%)				
Latino or Hispanic	4 (10%)	2 (9%)	2 (12%)	2 (18%)
Not Latino or	35 (90%)	21 (91%)	14 (82%)	9 (82%)
Hispanic				
Unknown	0 (0%)	0 (0%)	1 (6%)	0 (0%)
Race, n(%)				
Black or African-American	3 (8%)	2 (9%)	6 (35%)	4 (36%)
Asian	2 (5%)	1 (4%)	1 (6%)	0 (0%)
White	30 (77%)	18 (78%)	9 (53%)	7 (64%)
Multiple races	4 (10%)	2 (9%)	1 (6%)	0 (0%)
Behavioral scores, mean (SD)				
NVDQ	115.70 (13.81)	113.67 (12.86)	88.41 (19.58)	85.25 (16.62)
VDQ	99.84 (10.63)	108.60 (12.86)	46.77 (16.06)	76.64 (23.19)
Vineland-II Socialization	101.81 (6.05)	105.55 (7.19)	86.35 (6.13)	84.38 (9.81)
ASD diagnosis, n(%)		0 (0%)		7 (41%)

*NVDQ* Non-Verbal Developmental Quotient*. VDQ* Verbal Developmental Quotient

### Procedures

The study design was longitudinal, comprising study visits at 12 and/or 18 months (depending on age of study entry), 24 months, and 36 months. At each study visit, participants received a neurodevelopmental assessment supervised by doctoral-level clinicians, including the MSEL and Vineland Adaptive Behavior Scales, 2^nd^ Edition [31]. Two scores were computed from the MSEL and used in the current analysis: the Verbal Developmental Quotient (VDQ; using the Receptive and Expressive subscales) and the Nonverbal Developmental Quotient (NVDQ; using the Fine Motor and Visual Reception subscales). DQs are analogs of IQs and are calculated by averaging the age equivalents from each subscale, dividing by the chronological age, and multiplying the result by 100. From the Vineland-II, the Socialization standard score was used to reflect a continuous metric of social skills relative to normative expectations. The Socialization score is norm-referenced with a population mean of 100 and SD of 15.

**Fig. 1:**
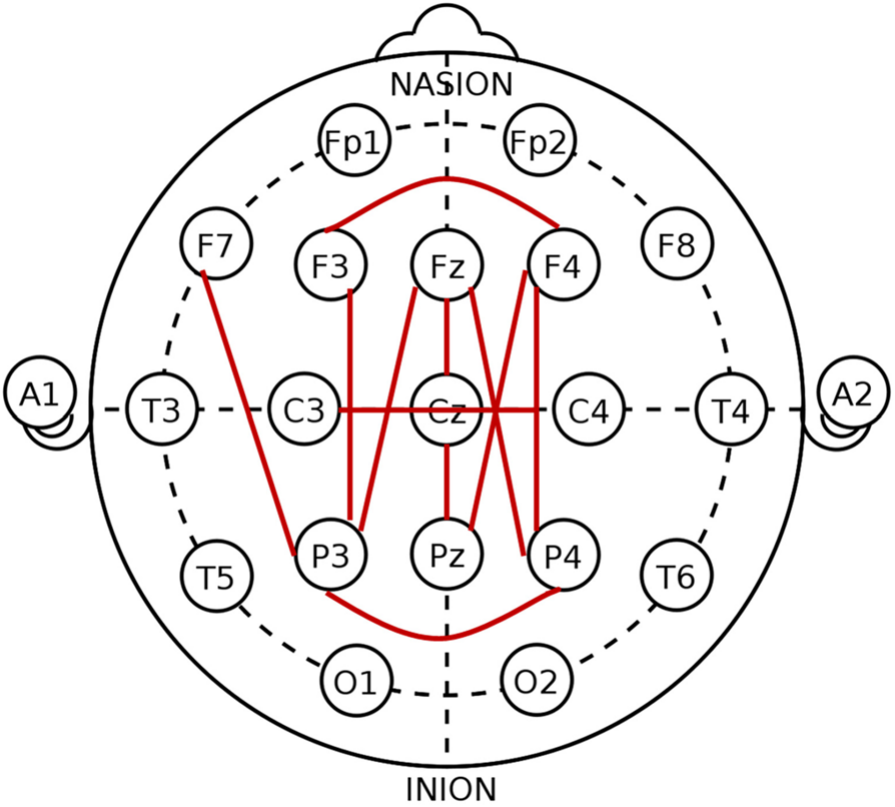
Electrode placement. Red lines indicate electrode pairs pulled for coherence analysis

An optional polysomnogram was performed at the participants’ first visit (baseline; BL) and at the 36-month visit (follow-up; FU); those with valid sleep EEG data at either visit were included in the current analysis. A total of 34 (LangD, n=11; TD, n=23) participants had both BL and FU sleep studies, while 21 (LangD, n=5; TD, n=16) had only BL. Digital EEGs were recorded during awake, drowsy, and sleep states using the 10-20 system of electrode placement (Fig. 1).

### Analysis

#### EEG Coherence

Clean 10-minute segments of awake, slow-wave sleep (SWS), and rapid eye movement (REM) sleep were selected for coherence analysis, which was performed masked to participant group using Neuroguide software (Applied Neuroscience Inc., St. Petersburg, FL) with a linked-ear montage.

Electrode pairs (F3-P3, F4-P4, F4-Pz, P3-Fz, P4-Fz, P3-F7, C3-C4, Cz-Pz, F3-F4, Fz-Cz, and P3-P4; Fig. 1) were selected for coherence analysis based on findings from Buckley et al. [32] which found increased coherence in children with ASD compared to typically developing controls, concentrated in frontal-parietal pairs. Frequency bands selected for coherence analysis are Delta (1-4 Hz), Theta (4-8 Hz), Alpha 1 (8-10 Hz), Alpha 2 (10-12 Hz), Beta 1 (12-15 Hz), Beta 2 (15-18 Hz), and Beta 3 (18-25 Hz). Analysis was performed across three sleep stages (awake, REM, SWS), eleven electrode pairs, and seven frequency bands, yielding 231 models.

Coherence values were subjected to Fisher transformation and fixed effects of group (LangD versus TD) and age in months (centered at 18 months) were evaluated with the lme4 [26] package for R version 4.2.1 [33] using a linear mixed model with a random subject-level intercept. These models addressed the questions of whether coherence depended on group, age, or both. False detection rate (FDR) p-value correction was applied to these effects (693 tests in total: three effects within each of 231 models). In separate exploratory analyses, we evaluated the relationship between coherence and behavioral features (MSEL VDQ and NVDQ, and Vineland-II Socialization standard score) by adding the mean-centered behavioral score as a fixed effect. Because these variables are aliased with group membership, we included an interaction between group and behavioral variable, and evaluated the slope of the behavioral variable on coherence within each group to avoid confounding. The parameters of interest in these models were the simple slopes of the behavioral feature within each group. FDR p-value correction was applied to the list of these slopes (462 tests).

#### Spindle Properties

Both slow spindles (9 Hz and 11 Hz) and fast spindles (13 Hz and 15 Hz) were evaluated separately for density (number of spindles per minute), amplitude, duration, frequency (Hz), and chirp (intra-spindle frequency change). Spindle data were analyzed from all 16 channels using Luna (http://zzz.bwh.harvard.edu/luna/), a software package developed at Harvard Medical School designed to analyze polysomnogram recordings and automate spindle analysis. The Luna software utilized a Morlet wavelet transformation method of spindle detection. Spindle frequency, amplitude, duration, and epoch counts were recorded for each spindle and plotted on a graph for visual analysis and verification.

The effect of age on spindle features was evaluated using linear mixed effects models with fixed effects for study visit (baseline, BL vs. follow-up, FU), group, and sex, and a random subject-level intercept. Differences between LangD and TD group were tested using logistic regression where diagnostic status was a dependent variable and spindle characteristics at each channel as an independent variable with age and sex added as covariates. FDR p-value correction was applied separately for two sets of the analyses: BSL vs FU with mixed effect regression (320 tests in total – 16 channels x 5 spindle metrics x 4 spindle frequencies); LangD vs TD with logistic regression (528 tests in total – 16 channels x 11 spindle metrics at spindle frequencies were significant effect between BSL and FU was observed x 3 conditions BSL, FU, FU-BSL). The relationship of spindle density to behavioral scores were evaluated as described above for coherence.

For all analyses, the p-values were corrected using the FDR method as described above. To make the large number of statistical tests tractable for presentation, we use a graphical approach. The complete set of uncorrected and corrected p-values and test statistics is available as a supplement.

## Results

### Coherence

**Fig. 2:**
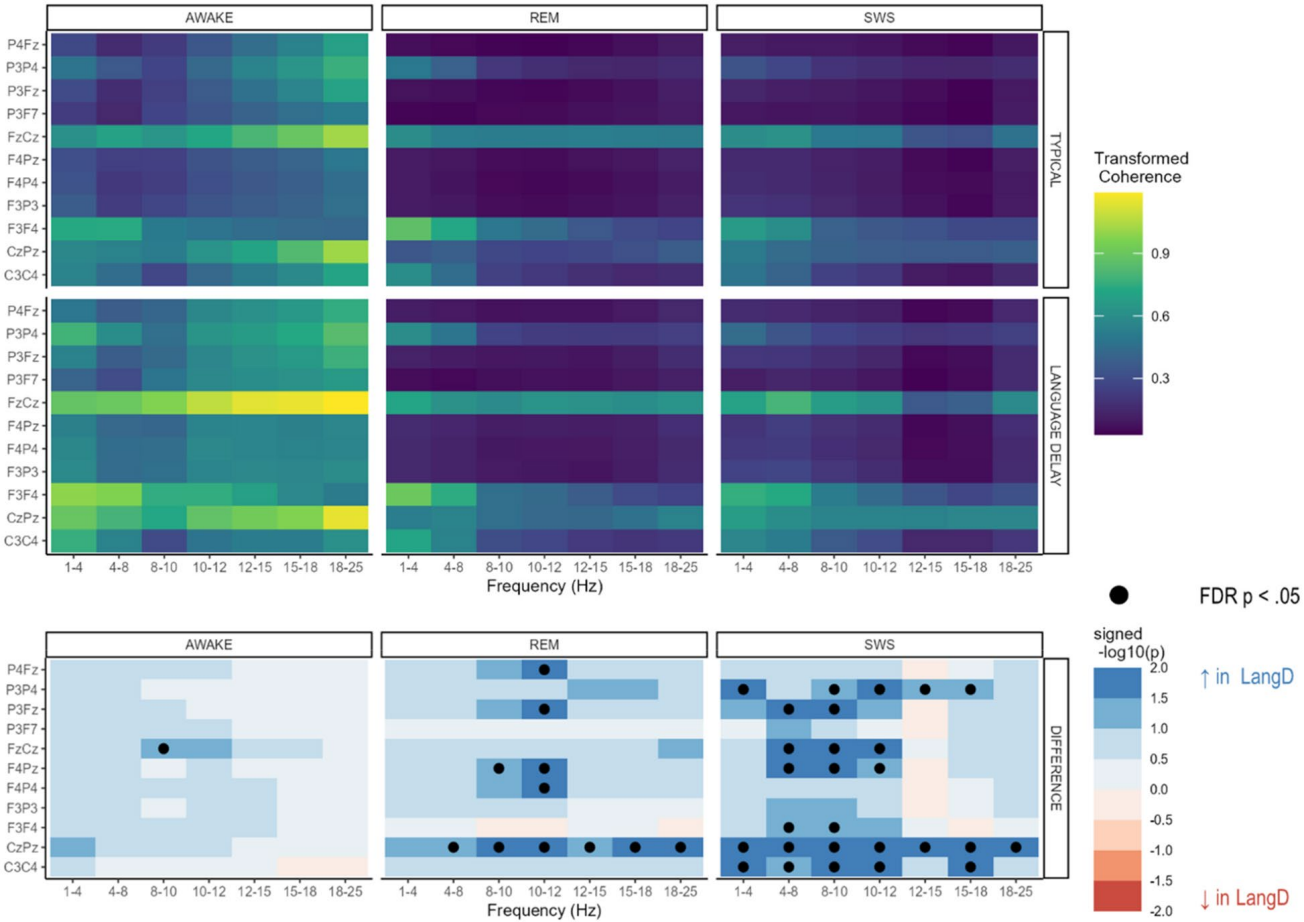
Model-estimated mean coherence at 18 months in the typical group (top row), language delay group (middle row), and the difference between these estimates (bottom row)

Group differences in coherence during awake, REM, and SWS stages are shown in Fig. 2. Increased coherence was observed for LangD relative to TD, with differences being most prominent during SWS. Few differences in coherence between LangD and TD were observed during the awake state. The group-by-age interactions indicated that the effects of group on coherence did not depend on age.

**Fig. 3:**
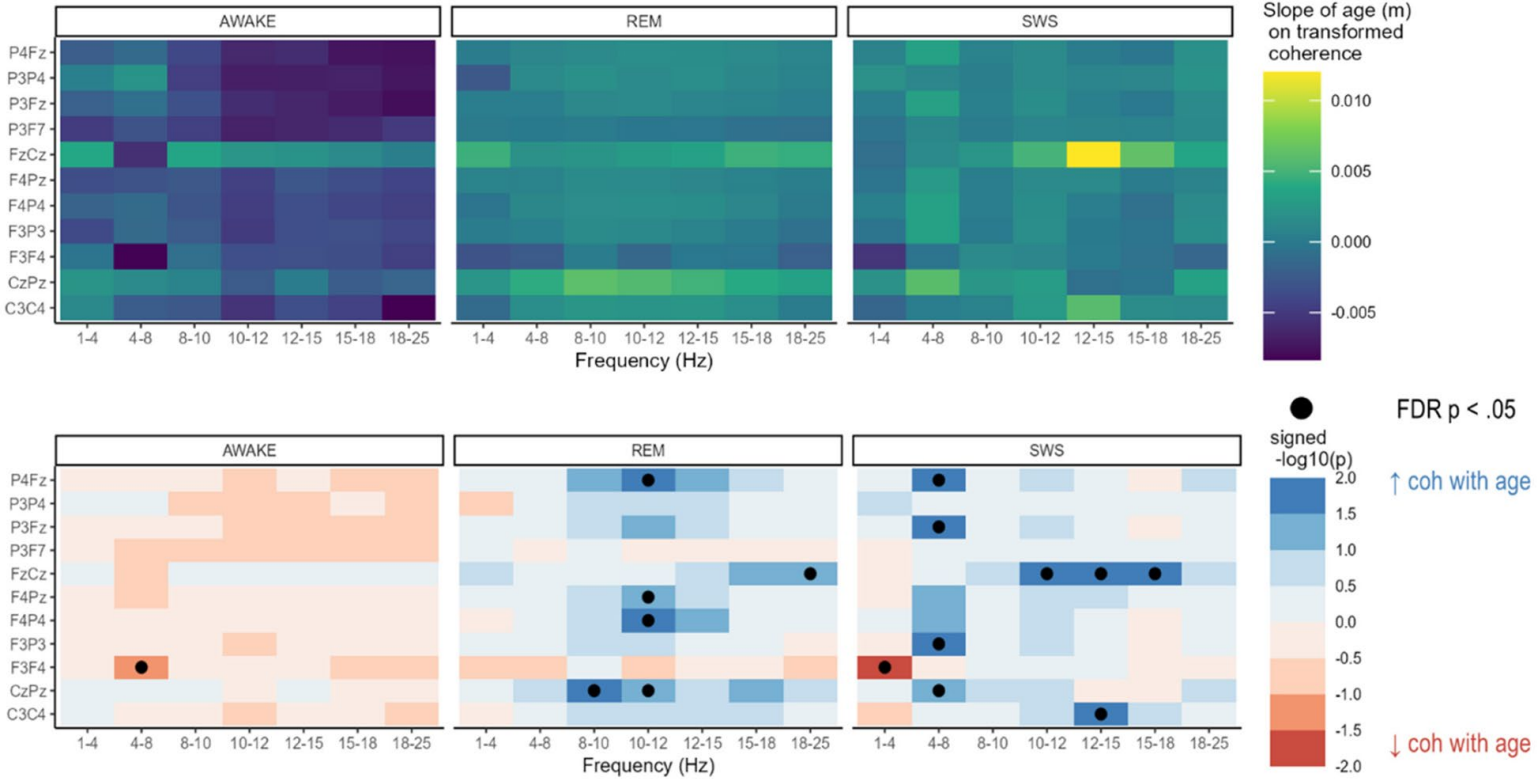
Model-estimated slope of age on coherence in the full sample (top row) and FDR-corrected p-values for those slopes

The effect of age on coherence is illustrated in Fig. 3. Increased coherence with age was found for four electrode pairs (C3-C4, F4-Pz, Fz-Cz, and P3-Fz), all within the beta 1 frequency (12-15 Hz). Decreased coherence with age was only found for one frequency/pair combination, F3-F4 in the delta frequency.

**Fig. 4:**
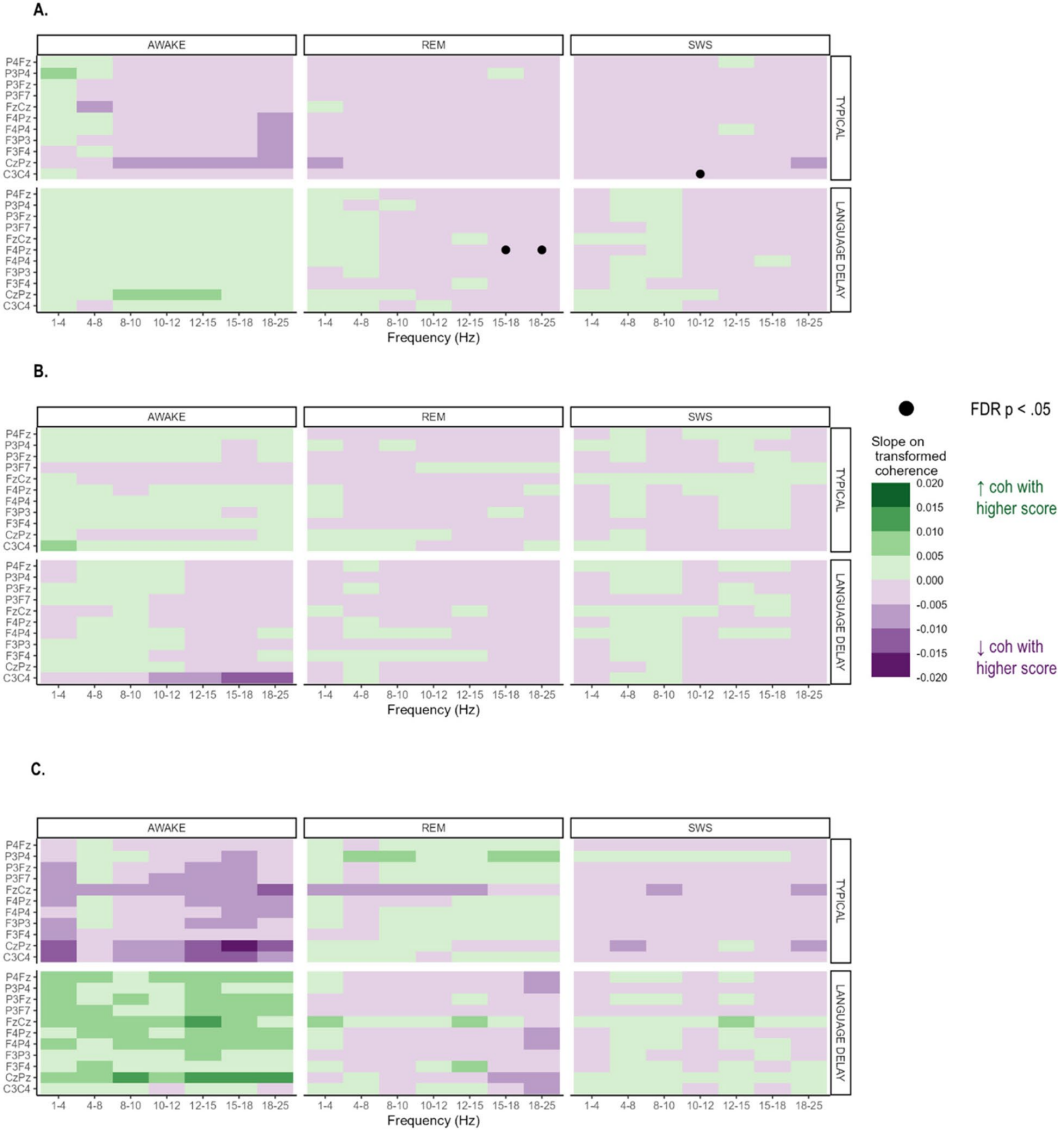
Simple slopes of VDQ (Panel A), NVDQ (Panel B), and Socialization (Panel C) on coherence. FDR-corrected p-values < .05 are marked with a black circle

In an exploratory analysis, within-group relationships between coherence and behavioral scores (NVDQ, VDQ, and Socialization) were evaluated in separate models. Very few significant relationships between scores and coherence were found for VDQ, NVDQ, or socialization (Fig. 4).

### Spindle Properties

#### Density

**Fig. 5:**
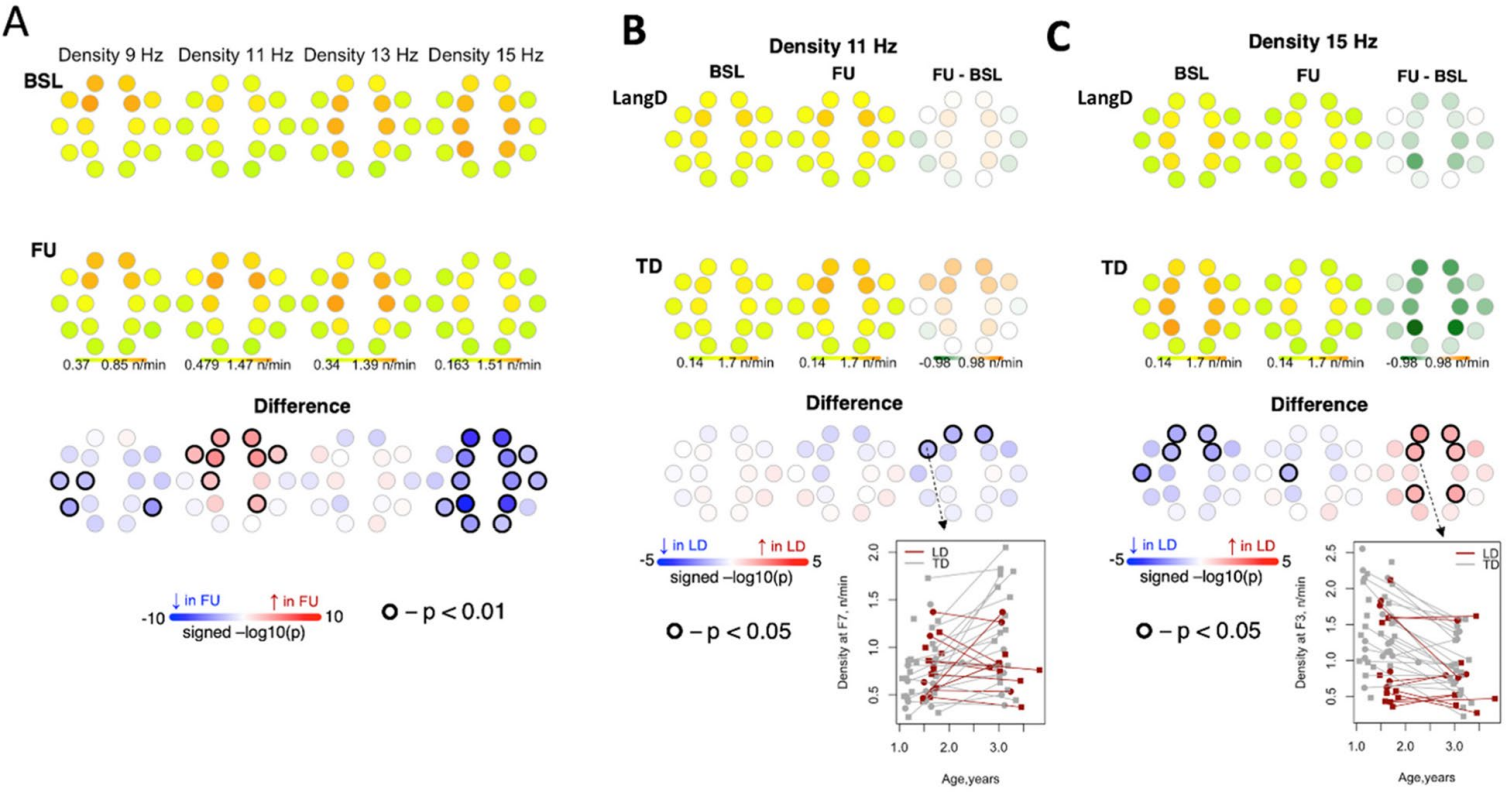
Density of spindles between baseline and follow-up visits. Panel A shows density of spindles between baseline and follow-up for the entire sample. Panel B shows differences in changes in density from baseline to follow-up between LangD and TD for 11 Hz spindles. Panel C shows differences in changes in density from baseline to follow-up between LangD and TD for 15 Hz spindles

In the combined sample, density of slow spindles at 9 Hz *decreased* from BL to FU in central and temporal channels. Density of slow spindles at 11 Hz *increased* from BL to FU in frontal channels (Fig. 5). This increase was greater for TD compared to LangD. Furthermore, density of fast spindles at 15 Hz *decreased* from BL to FU across multiple channels (Fig. 5). At 15 Hz, spindle density was lower for LangD than TD in frontal channels at BL; this difference was smaller in magnitude at FU (Fig. 5).

As an exploratory analysis, we evaluated the relationship of NVDQ, VDQ, and Socialization to density within each study group. The parameter estimates tended to be negative, associating higher density with lower behavioral scores, but the confidence intervals were wide and contained zero. The single exception was for the relationship of Socialization in the LangD group to spindle density in 15 Hz at the T6 electrode.

#### Duration

**Fig. 6:**
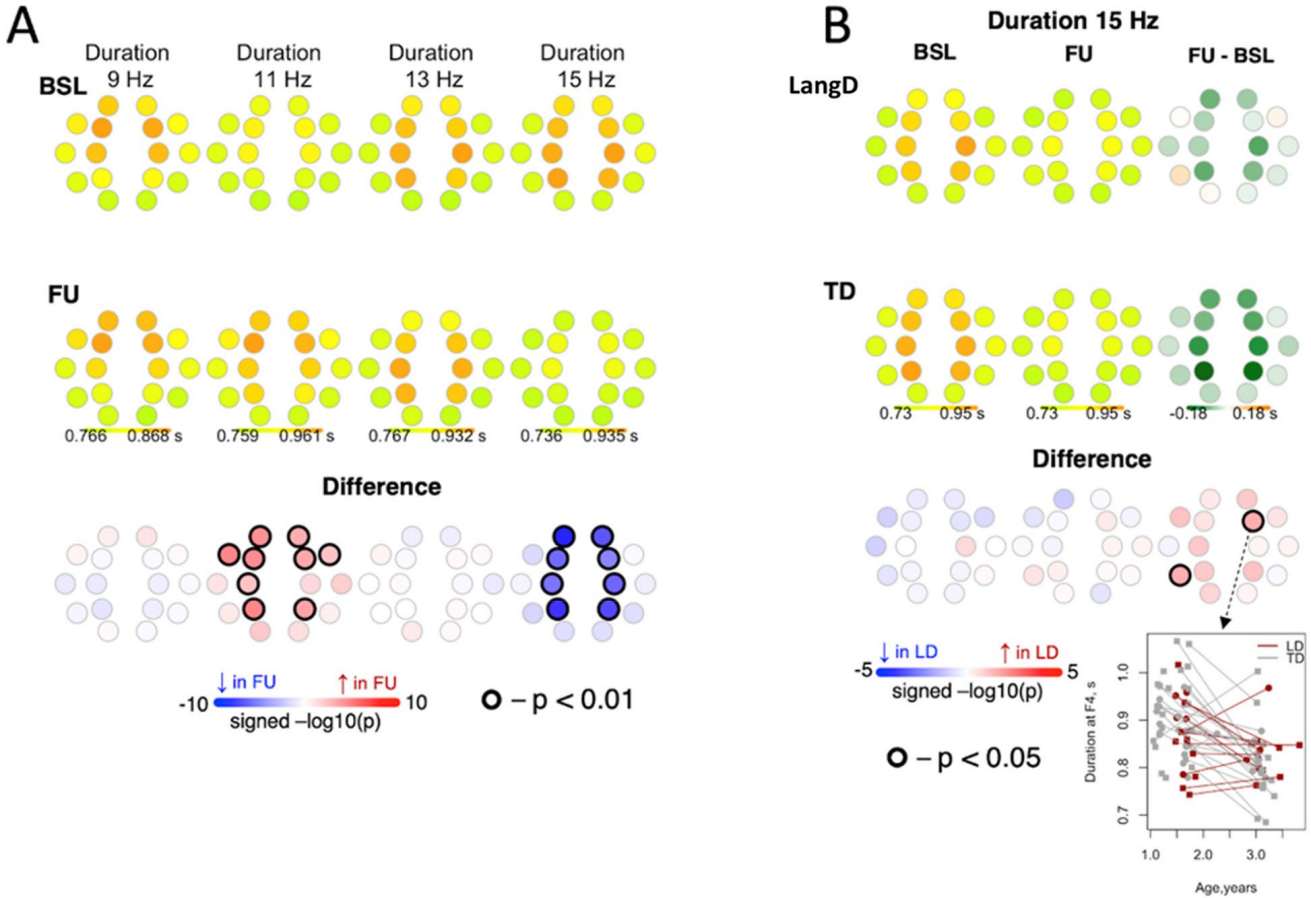
Differences in duration of spindles between baseline and follow-up. Panel A shows differences in duration of spindles between baseline and follow-up for the entire sample. Panel B shows differences in changes in duration from baseline to follow-up between LangD and TD for 15 Hz spindles

In the combined sample, duration of slow spindles at 11 Hz *increased* from BL to FU across multiple channels, whereas duration of fast spindles at 15 Hz *decreased* from BL to FU across multiple channels (Fig. 6). This decrease was lower in magnitude for LangD compared to TD.

#### Frequency

**Fig. 7:**
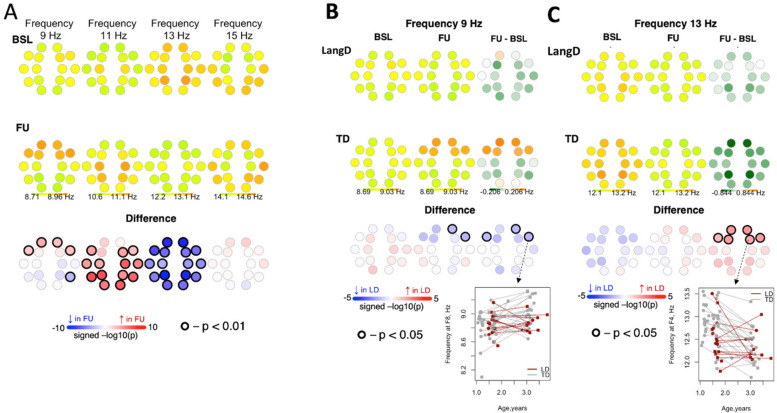
Differences in frequency of spindles between baseline and follow-up. Panel A shows differences in frequency of spindles between baseline and follow-up for the entire sample. Panel B shows differences in frequency from baseline to follow-up between LangD and TD for 9 Hz spindles. Panel C shows differences in frequency from baseline to follow-up between LangD and TD for 13 Hz spindles

In the combined sample, frequency of slow spindles at 11 Hz *increased* from BL to FU in posterior channels. Frequency of slow spindles at 9 Hz increased from BL to FU in frontal channels, and this increase was lower in magnitude for LangD compared to TD (Fig. 7) Frequency of fast spindles at 13 Hz *decreased* from BL to FU across all channels. This decrease was lower in magnitude for LangD compared to TD.

#### Chirp

**Fig. 8:**
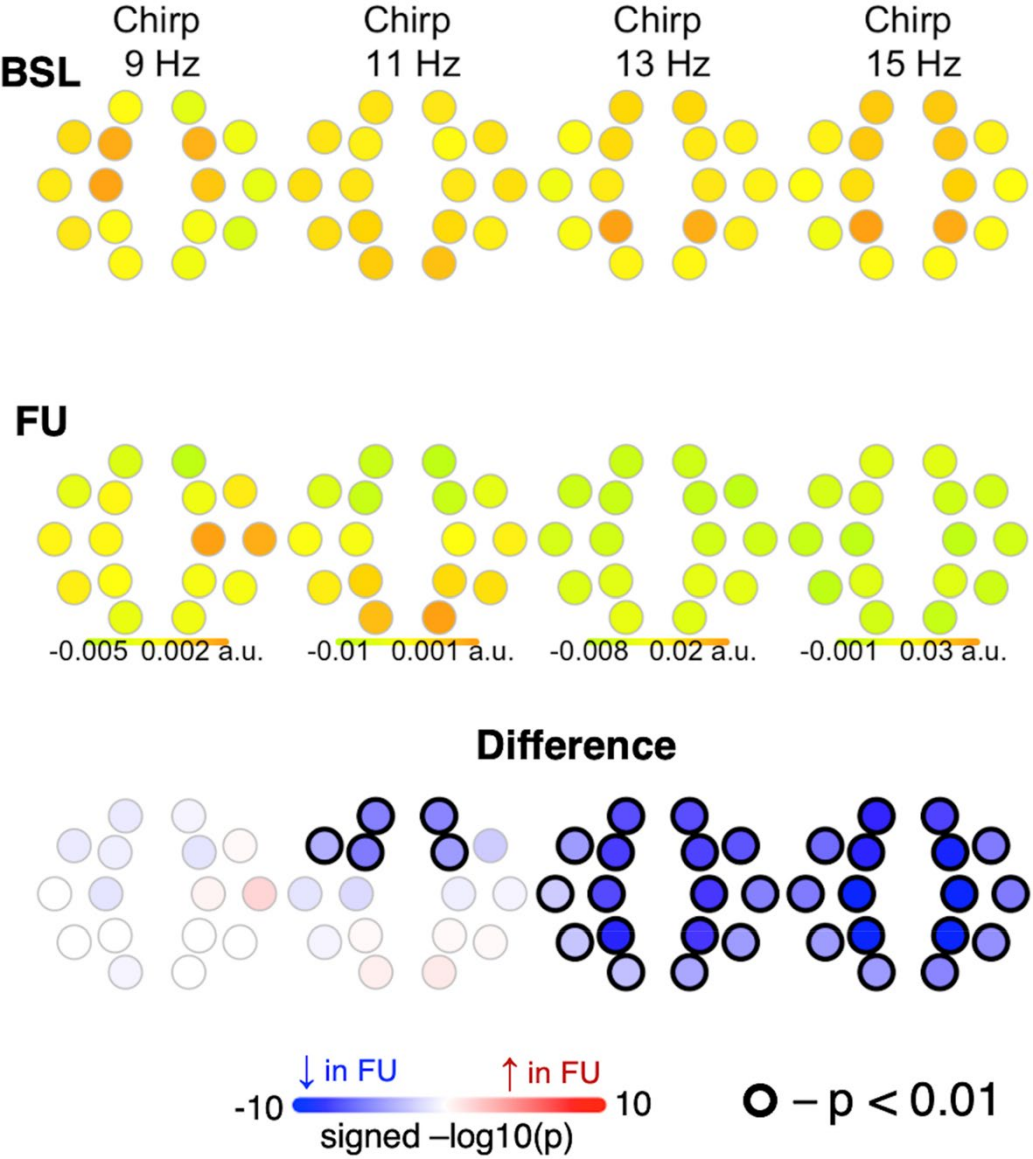
Differences in chirp (intra-spindle frequency change) from baseline to follow-up

Chirp of slow spindles at 11 Hz and fast spindles at 13 and 15 Hz decreased from BL to FU, with no difference between TD and LangD (Fig. 8).

## Discussion

We investigated EEG coherence and sleep spindle properties at two time points in toddlers with and without language delay. Increased coherence found for toddlers with receptive/expressive language delay was state- and stage-dependent, with differences most prominent during SWS in central electrode pairs across frequency bands. The group differences did not depend on age, indicating that any differences between LangD and TD may have already been present prior to toddler age. In exploratory analyses, few associations between continuous behavioral variables and coherence were observed within-group. This suggests that coherence differences are not dimensionally related to severity of neurodevelopmental impairments. An effect of age was observed on spindle properties for both groups; however, the LangD group demonstrated a smaller magnitude of change in spindle density, duration, and frequency in comparison to the TD group.

Interestingly, most of the differences in coherence between LangD and TD were detected in the alpha frequency bands. The alpha band has been implicated in research on neurodevelopmental differences. A previous study by Orekhova et al. [34] found alpha range hyper-connectivity in 14-month-old high-risk infants who were later diagnosed with ASD at 3 years old, suggesting that alpha hyper-connectivity may be an important attribute of ASD neurophysiology. Previous research has indicated that alpha oscillations detected by scalp EEG may contain signals from thalamocortical interactions [35], which has been speculated by Linke et al. [36] to play a crucial role in sleep disruptions in ASD. Alpha oscillations also appear to play an important role in language development and have been found to be correlated with typically developing children’s oral language abilities [35]. In this sample, differences in alpha-frequency coherence between LangD and TD may indicate abnormal thalamocortical functioning in LangD children with increased likelihood for neurodevelopmental disability and may also play a role in differences in language skill and trajectory of attainment.

This was an exploratory analysis intended to generate hypotheses for future confirmatory study, especially in larger samples that will allow for correlation of early sleep EEG characteristics with later diagnostic outcomes. Given that differences in EEG coherence were most prominent during SWS in our sample, future research may be more efficient in focusing primarily on SWS. Furthermore, given our findings regarding differences in sleep spindle properties, future research could investigate whether specific alterations in spindle density, duration, and frequency may predict later neurodevelopmental outcomes, including ASD diagnosis. Although very early ascertainment for later neurodevelopmental differences would be a challenge and require a larger sample size than in the current study, longitudinal analyses encompassing both infancy and childhood would allow for a more developmentally informed understanding of trajectories in coherence and spindle differences. Investigating whether these differences remain stable from early childhood to adulthood would also be illuminating and add to the literature finding cross-sectional differences in older children and adolescents with and without neurodevelopmental diagnoses like ASD.

The current results must be interpreted with respect to several limitations. This was a small study, meaning the power to detect small differences was low, especially given the multiplicity of tests. As such, prospective evaluation of these exploratory results is necessary. The LangD group was ascertained based on both receptive and expressive language delay, which placed them at increased risk of poorer outcomes [29]. Indeed, seven (44%) did go on to receive an ASD diagnosis before the end of study participation. We could not locate epidemiological data with which to compare this rate of ASD diagnosis among children with receptive and expressive language delay in toddlerhood, but it is consistent with increased rates of poorer educational and employment outcomes in later childhood and early adulthood [37]. Regardless, this subset of children who went on to receive an ASD diagnosis was too small for group-level comparison to the remainder of the LangD sample and the TD group, indicating the need for a larger initial sample size.

We note that the inclusion criteria dictated that the LangD sample would have uniformly lower scores on developmental testing than the TD group. However, the TD group in this sample had above-average developmental scores. This may be a function of recruitment practices (e.g., geographical location), but it may also be true that parents with mild concerns would be unlikely to respond to recruitment materials, shifting the average developmental scores in those who did respond. Further, the TD group was on average 2.5 months younger than LangD group at baseline, which could represent a meaningful difference in such young children. These age differences were addressed statistically, but future research with closer age matching may yeild more valid conclusions.

Although the longitudinal nature of this study is a significant strength, there were only two time points and some attrition. We assumed that this missingness was noninformative (i.e., not related to the concepts under study) and that it did not bias our results, but future research with more timepoints and complete data will improve the validity of interpretation. Finally, we note that the study design required that our exploration of dimensional correlates of the EEG parameters be performed within each study group, to guard against errors of inference. However, this reduced the functional sample size and therefore the power of these analyses to detect effects. Future analyses in larger samples that were not ascertained based on the developmental correlates will provide a better test of these questions.

## Conclusions

In conclusion, our study of toddlers at risk of later neurodevelopmental diagnoses due to their receptive/expressive language delay reveals early differences in sleep unique electrophysiologic signatures, specifically spindle properties and sleep EEG coherences, which may potentially serve as biomarkers for neurodevelopmental risk. These findings underscore the significance of investigating sleep-related brain activity to understand language development and neurodevelopmental disorders. Sleep EEG presents a promising avenue to elucidate neural connectivity patterns during critical developmental stages, offering unique insights into the functional maturation of brain circuits that influence language processing and cognitive development.

By identifying potential markers for neurodevelopmental differences, our study also highlights the importance of early detection and monitoring. The state- and stage-dependent increases in EEG coherence, particularly during slow-wave sleep, emphasize the relevance of sleep-related brain activity as a window into neural connectivity underlying language delay. Longitudinal assessments indicate that certain neurophysiological differences may be present as early as 18 months, supporting the value of early intervention for children at risk for neurodevelopmental disorders. Nevertheless, given the exploratory nature of this study and small sample size, further research with larger cohorts is necessary to validate and expand on these results. By unraveling the complexities of sleep-related brain activity, we gain insights into the neural substrates shaping language development and neurodevelopmental trajectories, facilitating targeted interventions and support strategies for optimal developmental outcomes.

## Supplementary Information

Supplementary Material 1.

Supplementary Material 2.

Supplementary Material 3.

## Data Availability

The data that support the findings of this study are available from the corresponding author upon reasonable request.

## Abbreviations

EEG: electroencephalogram
TD: typically developing
LangD: language delay
REM: rapid-eye movement
ASD: autism spectrum disorder
fMRI: functional magnetic resonance imaging
NREM: non-rapid-eye movement
SWS: slow-wave sleep
NIH: National Institutes of Health
MSEL: Mullen Scales of Early Learning
DSM-5: Diagnostic and Statistical Manual of Mental Disorders, Fifth Edition
VDQ: Verbal Developmental Quotient
NVDQ: Non-Verbal Developmental Quotient
DQ: Developmental Quotient
IQ: Intelligence Quotient
BL: baseline
