# Supplementary material for: Differences in sleep EEG coherence and spindle metrics in toddlers with and without receptive/expressive language delay: a prospective observational study
Source: J Neurodev Disord. 2025 Feb 22;17:11. doi: 10.1186/s11689-024-09586-1 (PMC11847392; doi:10.1186/s11689-024-09586-1)
Supplement: Supplementary file 1 — Supplementary Material 1. [file 11689_2024_9586_MOESM1_ESM.docx]

Table # Standardized b-coefficients and p-values of differences between baseline and follow-up in spindle parameters

| **VAR** | **FRQ** | **C3**  **b-coef p-val** | **C4**  **b-coef p-val** | **F3**  **b-coef p-val** | **F4**  **b-coef p-val** | **F7**  **b-coef p-val** | **F8**  **b-coef p-val** | **Fp1**  **b-coef p-val** | **Fp2**  **b-coef p-val** | **O1**  **b-coef p-val** | **O2**  **b-coef p-val** | **P3**  **b-coef p-val** | **P4**  **b-coef p-val** | **T3**  **b-coef p-val** | **T4**  **b-coef p-val** | **T5**  **b-coef p-val** | **T6**  **b-coef p-val** |
| --- | --- | --- | --- | --- | --- | --- | --- | --- | --- | --- | --- | --- | --- | --- | --- | --- | --- |
|  |  |  |  |  |  |  |  |  |  |  |  |  |  |  |  |  |  |
| **Density** | **9** | -3.2  3e-03 | -2.6  1e-02 | -1.8  9e-02 | -2.3  3e-02 | -2.1  4e-02 | -2.7  1e-02 | -0.6  6e-01 | 0.8  4e-01 | -2.5  2e-02 | -1.8  8e-02 | -1.8  8e-02 | -1.5  1e-01 | -3.1  4e-03 | -2.7  1e-02 | -3.9  4e-04 | -4.6  8e-05 |
|  | **11** | 3  6e-03 | 2.3  3e-02 | 4.6  6e-05 | 4.4  1e-04 | 3.2  3e-03 | 2.8  8e-03 | 3.9  4e-04 | 4.3  2e-04 | -0.9  4e-01 | 0  1e+00 | 2.5  2e-02 | 3.3  2e-03 | -0.9  4e-01 | -1.4  2e-01 | -0.8  5e-01 | -0.5  6e-01 |
|  | **13** | 1.3  0.20 | 1.4  0.16 | 0.1  0.91 | 0.9  0.36 | 1.6  0.12 | 0.6  0.57 | -2.1  0.05 | -2.5  0.02 | -0.6  0.57 | 1.5  0.16 | -2.4  0.02 | -0.6  0.58 | -1.6  0.11 | -2  0.05 | -0.8  0.46 | 0.5  0.61 |
|  | **15** | -4.3  2e-04 | -4.6  7e-05 | -5.1  2e-05 | -5.1  2e-05 | -2.6  1e-02 | -2.9  7e-03 | -7.4  2e-08 | -6.7  1e-07 | -4.3  2e-04 | -3  5e-03 | -8.8  5e-10 | -7  7e-08 | -2.7  1e-02 | -3.4  2e-03 | -3.5  2e-03 | -2.5  2e-02 |
| **Duration** | **9** | -1.5  0.1 | -1.2  0.2 | -0.3  0.8 | -0.4  0.7 | 0.9  0.4 | 0.5  0.6 | 1.3  0.2 | 1.6  0.1 | 0.6  0.6 | -0.6  0.5 | -1.6  0.1 | -1.1  0.3 | -1.4  0.2 | -0.3  0.8 | -0.8  0.4 | -0.4  0.7 |
|  | **11** | 2.9  6e-03 | 2.2  4e-02 | 4.6  8e-05 | 3.9  5e-04 | 4.8  5e-05 | 3  6e-03 | 4.4  1e-04 | 3.6  1e-03 | 2.7  1e-02 | 1.9  6e-02 | 4.8  4e-05 | 3.9  5e-04 | 1.6  1e-01 | 2.5  2e-02 | 1.3  2e-01 | 1.3  2e-01 |
|  | **13** | 0.3  0.8 | -0.7  0.5 | -0.6  0.5 | 0.4  0.7 | 0.7  0.5 | 0.4  0.7 | -1.3  0.2 | -1  0.3 | 1.5  0.1 | 0.6  0.6 | 0  1.0 | 0.6  0.5 | 0.2  0.9 | -1.3  0.2 | -0.2  0.9 | -0.2  0.8 |
|  | **15** | -5.5  6e-06 | -5.8  2e-06 | -5.7  3e-06 | -5  2e-05 | -2.1  4e-02 | -0.6  5e-01 | -8.2  3e-09 | -6.3  5e-07 | -2  5e-02 | -1.9  6e-02 | -7.4  2e-08 | -6.7  2e-07 | -1  3e-01 | -1.3  2e-01 | -2.3  3e-02 | -1.9  7e-02 |
| **Frequency** | **9** | -0.6  0.551 | -1.7  0.096 | 1.2  0.231 | 1.3  0.190 | 2.8  0.008 | 3  0.005 | 3.6  0.001 | 3  0.005 | -1  0.323 | -0.5  0.630 | -1.8  0.084 | -2  0.050 | -0.4  0.697 | -0.9  0.378 | -1.1  0.273 | -3.2  0.003 |
|  | **11** | 3.8  6e-04 | 5.9  2e-06 | 2.7  1e-02 | 4  4e-04 | 3.9  5e-04 | 3  5e-03 | 2.6  2e-02 | 0.9  4e-01 | 5.1  2e-05 | 5.8  2e-06 | 6.3  5e-07 | 4.5  9e-05 | 4.1  3e-04 | 4.7  5e-05 | 6.2  7e-07 | 5.7  3e-06 |
|  | **13** | -3.7  9e-04 | -4.4  1e-04 | -5.7  3e-06 | -5.4  7e-06 | -6.7  2e-07 | -5.6  4e-06 | -8.1  4e-09 | -8.5  1e-09 | -6.1  9e-07 | -5.5  5e-06 | -9.8  5e-11 | -8.7  8e-10 | -4.6  8e-05 | -4.6  6e-05 | -5.2  1e-05 | -5.5  6e-06 |
|  | **15** | 0.5  0.65 | -0.5  0.62 | 1.2  0.24 | 0.2  0.87 | -0.3  0.77 | 0.9  0.36 | 1.8  0.08 | 2.5  0.02 | -0.5  0.60 | -1.8  0.09 | 0.3  0.75 | 0.6  0.56 | 0.9  0.35 | 1.7  0.10 | 0.3  0.78 | 0.5  0.65 |
| **CHIRP** | **9** | -1.7  0.11 | 0.7  0.47 | -1.2  0.25 | -1.7  0.10 | -1.4  0.16 | 0.6  0.56 | -1.5  0.15 | -0.9  0.40 | -0.8  0.43 | 0.1  0.94 | -0.2  0.86 | -0.1  0.90 | 0.1  0.89 | 2.4  0.02 | -0.2  0.85 | 0.2  0.82 |
|  | **11** | -2.1  5e-02 | -1.2  2e-01 | -5.2  1e-05 | -4.3  2e-04 | -3.7  8e-04 | -2.7  1e-02 | -5.1  2e-05 | -5  2e-05 | 1  3e-01 | 1.5  2e-01 | 0.3  7e-01 | 0.5  6e-01 | -1.7  9e-02 | -0.9  4e-01 | -0.8  5e-01 | 0.3  8e-01 |
|  | **13** | -6.8  1e-07 | -7.2  4e-08 | -7  8e-08 | -6.8  1e-07 | -4.3  2e-04 | -6.4  4e-07 | -6.6  2e-07 | -6.5  3e-07 | -3.1  4e-03 | -4  4e-04 | -8.2  3e-09 | -7.2  4e-08 | -2.8  8e-03 | -5.1  1e-05 | -2.8  8e-03 | -4.3  1e-04 |
|  | **15** | -10  3e-11 | -11.1  2e-12 | -8.1  4e-09 | -8.8  6e-10 | -5.7  3e-06 | -5.3  9e-06 | -8  5e-09 | -7  9e-08 | -4.3  2e-04 | -5  2e-05 | -12.9  5e-14 | -11.9  4e-13 | -5.3  8e-06 | -5.2  1e-05 | -4.3  2e-04 | -4.6  7e-05 |

Table # Standardized b-coefficients and p-values of differences between LD and TD groups in spindle parameters at baseline and follow-up and baseline – follow-up conditions

| **VAR** | **Cont**  **rast** | **C3**  **b-coef**  **p-val** | **C4**  **b-coef**  **p-val** | **F3**  **b-coef**  **p-val** | **F4**  **b-coef**  **p-val** | **F7**  **b-coef**  **p-val** | **F8**  **b-coef**  **p-val** | **Fp1**  **b-coef**  **p-val** | **Fp2**  **b-coef**  **p-val** | **O1**  **b-coef**  **p-val** | **O2**  **b-coef**  **p-val** | **P3**  **b-coef**  **p-val** | **P4**  **b-coef**  **p-val** | **T3**  **b-coef**  **p-val** | **T4**  **b-coef**  **p-val** | **T5**  **b-coef**  **p-val** | **T6**  **b-coef**  **p-val** |
| --- | --- | --- | --- | --- | --- | --- | --- | --- | --- | --- | --- | --- | --- | --- | --- | --- | --- |
|  |  |  |  |  |  |  |  |  |  |  |  |  |  |  |  |  |  |
| DENS_9 | BSL | -0.3  0.33 | -0.2  0.59 | -0.3  0.35 | -0.3  0.39 | -0.6  0.11 | -0.5  0.17 | -0.8  0.04 | -0.7  0.06 | -0.6  0.13 | -1  0.03 | -0.3  0.35 | -0.3  0.39 | -0.2  0.61 | -0.3  0.41 | -0.4  0.28 | -0.2  0.63 |
|  | FU | 0  0.9 | 0.6  0.2 | -0.1  0.9 | 0  1.0 | -0.2  0.6 | 0.1  0.9 | -0.6  0.3 | -0.4  0.4 | 0.3  0.4 | 0.1  0.7 | 0  1.0 | 0.3  0.5 | 0.2  0.6 | 0.4  0.3 | 0.3  0.5 | 0.4  0.4 |
|  | FU-BSL | -0.2  0.70 | 0.2  0.56 | -0.4  0.36 | -0.3  0.51 | -0.3  0.51 | -0.3  0.43 | -1.6  0.05 | -0.7  0.15 | 0.8  0.08 | 0.4  0.38 | 0.4  0.36 | 0.1  0.76 | 0  0.93 | 0.3  0.39 | 0.4  0.38 | 0.1  0.87 |
| DENS_11 | BSL | 0  0.9 | 0.1  0.7 | 0.1  0.8 | 0.1  0.7 | 0  0.9 | -0.2  0.6 | -0.2  0.5 | -0.1  0.7 | 0.1  0.8 | 0.1  0.8 | 0.2  0.6 | 0.2  0.6 | -0.1  0.7 | 0.3  0.4 | -0.2  0.6 | 0.3  0.4 |
|  | FU | -0.6  0.2 | 0.1  0.9 | -0.5  0.2 | -0.3  0.5 | -0.5  0.2 | -0.2  0.6 | -0.7  0.2 | -0.8  0.1 | 0.1  0.8 | 0.3  0.4 | -0.2  0.6 | 0  1.0 | 0  1.0 | 0.3  0.5 | 0.3  0.4 | 0.4  0.4 |
|  | FU-BSL | -0.5  0.29 | 0.1  0.88 | -0.8  0.16 | -0.6  0.23 | -1.4  0.04 | -1  0.07 | -1.3  0.04 | -1.7  0.03 | -0.3  0.50 | -0.2  0.67 | -0.2  0.64 | -0.2  0.59 | -0.8  0.10 | -0.3  0.42 | -0.4  0.32 | -0.5  0.27 |
| DENS_15 | BSL | -0.7  0.085 | -0.3  0.325 | -0.8  0.041 | -1  0.018 | -0.8  0.081 | -0.9  0.058 | -1  0.023 | -0.8  0.050 | -0.2  0.610 | -0.4  0.370 | -0.7  0.100 | -0.5  0.253 | -1.8  0.008 | -0.3  0.346 | -0.7  0.127 | -0.2  0.691 |
|  | FU | -1.1  0.05 | -0.5  0.26 | -0.4  0.32 | -0.4  0.29 | -0.2  0.53 | -0.1  0.80 | -0.2  0.60 | -0.2  0.65 | 0.1  0.90 | 0.4  0.35 | -0.7  0.15 | 0.1  0.79 | 0  0.96 | 0.1  0.75 | 0.2  0.62 | -0.1  0.82 |
|  | FU-BSL | 0.5  0.26 | 0.5  0.21 | 1.1  0.03 | 0.9  0.05 | 0.6  0.16 | 1  0.07 | 1.3  0.02 | 1.1  0.03 | 0.8  0.12 | 0.3  0.41 | 1.1  0.04 | 1.1  0.03 | 0.5  0.30 | 0.4  0.35 | 0.7  0.13 | 0.1  0.78 |
| DUR_11 | BSL | 0  0.95 | 0.3  0.49 | 0.5  0.17 | 0.3  0.41 | -0.2  0.66 | 0  0.94 | -0.2  0.60 | 0  0.97 | -0.4  0.30 | 0.4  0.29 | 0.1  0.74 | -0.1  0.74 | -0.7  0.07 | -0.1  0.86 | -0.4  0.24 | 0.1  0.86 |
|  | FU | -0.2  0.6 | 0.3  0.4 | 0  1.0 | 0.3  0.5 | -0.6  0.3 | -0.7  0.2 | -0.8  0.2 | -0.4  0.4 | -0.4  0.4 | 0.2  0.6 | -0.1  0.8 | -0.3  0.5 | -0.3  0.4 | -0.3  0.5 | 0.3  0.5 | -0.1  0.8 |
|  | FU-BSL | 0  0.98 | 0.2  0.59 | -0.3  0.50 | -0.2  0.68 | -1.1  0.06 | -0.6  0.23 | -0.7  0.16 | -0.6  0.19 | 0  1.00 | 0  0.97 | 0  0.91 | -0.2  0.65 | 0  0.94 | -0.2  0.59 | 0.4  0.38 | -0.2  0.57 |
| DUR_15 | BSL | 0  0.9 | 0.5  0.2 | -0.2  0.5 | -0.3  0.3 | -0.5  0.1 | -0.5  0.2 | -0.3  0.4 | -0.3  0.4 | -0.2  0.6 | -0.2  0.6 | -0.1  0.7 | -0.2  0.6 | -0.7  0.1 | 0  1.0 | -0.4  0.3 | 0.1  0.8 |
|  | FU | -0.2  0.60 | 0.2  0.61 | -0.1  0.73 | 0.3  0.41 | -0.4  0.33 | -0.2  0.61 | -0.8  0.09 | -0.1  0.76 | 0.1  0.75 | -0.4  0.33 | 0.3  0.53 | 0.2  0.72 | -0.1  0.82 | 0.2  0.69 | 0.4  0.34 | 0.1  0.85 |
|  | FU-BSL | 0.9  0.11 | 0.2  0.63 | 0.4  0.30 | 1.2  0.03 | 0.8  0.09 | 0.4  0.35 | 0.4  0.40 | 0.8  0.10 | 0.6  0.15 | 0.2  0.62 | 0.9  0.11 | 0.9  0.08 | -0.2  0.66 | 0.2  0.68 | 1.3  0.03 | -0.1  0.72 |
| FFT_9 | BSL | 0.4  0.3 | 0.6  0.1 | 0.5  0.3 | 0.3  0.5 | 0  0.9 | -0.1  0.7 | -0.2  0.7 | -0.2  0.5 | 0.6  0.1 | 0.1  0.9 | 0.2  0.6 | 0.4  0.4 | -0.1  0.8 | 0.4  0.3 | 0.1  0.8 | 0.2  0.5 |
|  | FU | 0  0.98 | -0.1  0.83 | -0.9  0.07 | -0.4  0.36 | -1  0.06 | -1.3  0.04 | -1.1  0.06 | -1.2  0.05 | 0  0.95 | -0.2  0.55 | 0.1  0.71 | -0.2  0.69 | 0.3  0.43 | 0  0.97 | 0.3  0.44 | 0.3  0.43 |
|  | FU-BSL | 0  0.99 | -0.1  0.91 | -1  0.05 | -0.7  0.11 | -1.1  0.05 | -1.2  0.04 | -0.6  0.19 | -1.1  0.05 | -1  0.07 | -0.5  0.30 | 0  0.97 | -0.1  0.83 | 0.1  0.90 | -0.5  0.26 | -0.1  0.74 | 0.1  0.78 |
| FFT_11 | BSL | 0  1.0 | 0.2  0.6 | -0.1  0.8 | -0.1  0.7 | 0.3  0.3 | 0.2  0.5 | 0.5  0.2 | 0  0.9 | 0.3  0.4 | 0.2  0.5 | 0.5  0.1 | 0.3  0.3 | 0.3  0.3 | 0.4  0.3 | 0.1  0.8 | -0.2  0.6 |
|  | FU | -0.2  0.7 | -0.1  0.7 | 0  0.9 | 0.1  0.9 | -0.3  0.4 | 0.2  0.7 | -0.2  0.6 | -0.1  0.8 | -0.6  0.2 | -0.4  0.3 | -0.4  0.4 | -0.7  0.1 | -0.5  0.2 | -0.6  0.2 | -0.6  0.2 | -0.5  0.2 |
|  | FU-BSL | 0.7  0.1 | 0.5  0.2 | 0.5  0.2 | 0.6  0.2 | 0.5  0.3 | 0.5  0.3 | 0.1  0.8 | 0.2  0.6 | -0.3  0.4 | -0.2  0.6 | 0.3  0.5 | -0.6  0.2 | 0  1.0 | 0.1  0.9 | 0.2  0.7 | -0.1  0.9 |
| FFT_13 | BSL | -0.3  0.44 | -0.3  0.39 | -0.6  0.13 | -0.6  0.09 | -0.5  0.17 | -0.6  0.09 | -0.4  0.20 | -0.5  0.20 | -0.1  0.71 | 0.2  0.56 | -0.7  0.07 | -0.3  0.39 | -0.8  0.05 | -0.3  0.33 | -0.6  0.12 | 0  0.95 |
|  | FU | -0.3  0.4 | -0.3  0.6 | 0.2  0.7 | 0  1.0 | 0  1.0 | 0.5  0.2 | 0.4  0.3 | 0.7  0.1 | 0  1.0 | 0.2  0.6 | -0.2  0.7 | 0.4  0.4 | -0.2  0.6 | 0.3  0.5 | 0  0.9 | 0  1.0 |
|  | FU-BSL | 0.3  0.47 | 0.3  0.42 | 1.5  0.02 | 1.3  0.03 | 1.1  0.04 | 1.5  0.02 | 1.3  0.01 | 1.4  0.01 | 0.6  0.19 | 0.6  0.17 | 0.8  0.08 | 0.8  0.07 | 0.6  0.18 | 0.6  0.18 | 0.8  0.08 | 0.2  0.61 |
| CHIRP_11 | BSL | 0  0.89 | 0.3  0.32 | 0.6  0.11 | -0.1  0.78 | -0.1  0.76 | -0.2  0.60 | 0.4  0.31 | 0  0.90 | 0.1  0.67 | -0.3  0.31 | 0.9  0.03 | 0.4  0.33 | -0.3  0.38 | 0.6  0.10 | -0.1  0.83 | 0  0.90 |
|  | FU | 0.3  0.42 | 0.4  0.28 | 0.7  0.12 | 0.8  0.11 | 0.7  0.13 | 0  0.99 | 0.7  0.12 | 0.4  0.34 | 0.1  0.73 | -0.1  0.77 | -0.8  0.07 | -0.4  0.36 | -0.1  0.83 | -0.5  0.29 | -0.2  0.66 | -0.8  0.11 |
|  | FU-BSL | 0  0.98 | -0.2  0.62 | 0.1  0.74 | 0.7  0.12 | 0.4  0.39 | 0  0.91 | 0.4  0.29 | 0.5  0.21 | 0.2  0.65 | 0.4  0.35 | -1.3  0.06 | -0.7  0.12 | 0.1  0.78 | -0.6  0.17 | -0.2  0.69 | -0.7  0.13 |
| CHIRP_13 | BSL | 0.2  0.6 | 0.3  0.4 | -0.1  0.7 | -0.2  0.6 | 0.3  0.5 | 0.3  0.5 | 0  0.9 | 0.1  0.8 | 0.4  0.2 | 0.3  0.4 | 0  1.0 | 0.3  0.4 | -0.3  0.4 | 0.3  0.4 | 0.1  0.7 | 0.5  0.2 |
|  | FU | -0.6  0.22 | -0.6  0.16 | 0  0.92 | 0  0.91 | 0.3  0.51 | 1.4  0.04 | 0.5  0.25 | 1  0.09 | -0.4  0.38 | -0.6  0.23 | -0.5  0.27 | 0.1  0.75 | 0.3  0.50 | 1  0.05 | 0.7  0.13 | 0.4  0.34 |
|  | FU-BSL | 0.2  0.65 | -0.2  0.62 | 0.9  0.11 | 0.6  0.18 | 0.3  0.40 | 1.1  0.06 | 0.9  0.08 | 0.9  0.07 | -0.1  0.76 | -0.4  0.37 | 0.6  0.21 | 0.3  0.54 | 0.3  0.40 | 0.9  0.08 | 0.2  0.67 | 0.6  0.20 |
| CHIRP_15 | BSL | -0.4  0.23 | -0.3  0.43 | -0.6  0.13 | -0.8  0.06 | 0.2  0.59 | -0.5  0.18 | -0.6  0.09 | -0.4  0.30 | -0.1  0.73 | 0  0.91 | -0.6  0.10 | -0.4  0.28 | -0.5  0.20 | -0.1  0.74 | -0.6  0.09 | -0.5  0.21 |
|  | FU | 0.2  0.5 | -0.7  0.2 | -0.4  0.3 | -0.3  0.5 | -0.2  0.6 | 0.1  0.8 | -0.1  0.8 | -0.3  0.5 | -0.1  0.7 | 0.2  0.7 | -0.5  0.3 | -0.1  0.8 | 0.4  0.4 | -0.3  0.4 | 0.4  0.4 | -0.2  0.7 |
|  | FU-BSL | 1.3  0.03 | 0.8  0.08 | 0.7  0.14 | 0.7  0.13 | -0.1  0.84 | 0.8  0.08 | 1.2  0.03 | 0  0.98 | 0.3  0.44 | 0  0.99 | 0.6  0.19 | 1  0.05 | 0.4  0.27 | 0.3  0.45 | 1.1  0.05 | 0.3  0.40 |
