# Supplementary material for: Differences in sleep EEG coherence and spindle metrics in toddlers with and without receptive/expressive language delay: a prospective observational study
Source: J Neurodev Disord. 2025 Feb 22;17:11. doi: 10.1186/s11689-024-09586-1 (PMC11847392; doi:10.1186/s11689-024-09586-1)
Supplement: Supplementary file 2 — Supplementary Material 2. [file 11689_2024_9586_MOESM2_ESM.docx]

Table Descriptions

Table #1 ParmsGroup. This table contains the fixed effects from the mixed model of coherence (dependent variable) as a function of group (LD versus TD), chronological age (centered at 18 months), and the age-by-group interaction. A separate model was specified for each combination of stage, electrode pair, and frequency.

Table #2 ParmsNVDQ. This table contains the fixed effects from the mixed model of coherence (dependent variable) as a function of group (LD versus TD), chronological age (centered at 18 months), NVDQ (centered at the sample mean), the NVDQ-by-group interaction, and the age-by-group interaction. A separate model was specified for each combination of stage, electrode pair, and frequency.

Table #3 ParmsVDQ. This table contains the fixed effects from the mixed model of coherence (dependent variable) as a function of group (LD versus TD), chronological age (centered at 18 months), VDQ (centered at the sample mean), the VDQ-by-group interaction, and the age-by-group interaction. A separate model was specified for each combination of stage, electrode pair, and frequency.

Table #4 ParmsSOC. This table contains the fixed effects from the mixed model of coherence (dependent variable) as a function of group (LD versus TD), chronological age (centered at 18 months), Socialization (centered at the sample mean), the Socialization-by-group interaction, and the age-by-group interaction. A separate model was specified for each combination of stage, electrode pair, and frequency.

Table #5 SlopesNVDQ. This table contains the simple slopes of NVDQ within each group (LD and TD) from the mixed model of coherence (dependent variable) as a function of group (LD versus TD), chronological age (centered at 18 months), NVDQ (centered at the sample mean), the NVDQ-by-group interaction, and the age-by-group interaction. A separate model was specified for each combination of stage, electrode pair, and frequency.

Table #6 SlopesVDQ. This table contains the simple slopes of VDQ within each group (LD and TD) from the mixed model of coherence (dependent variable) as a function of group (LD versus TD), chronological age (centered at 18 months), VDQ (centered at the sample mean), the VDQ-by-group interaction, and the age-by-group interaction. A separate model was specified for each combination of stage, electrode pair, and frequency.

Table #7 SlopesSOC. This table contains the simple slopes of Socialization within each group (LD and TD) from the mixed model of coherence (dependent variable) as a function of group (LD versus TD), chronological age (centered at 18 months), Socialization (centered at the sample mean), the Socialization-by-group interaction, and the age-by-group interaction. A separate model was specified for each combination of stage, electrode pair, and frequency.

Table #8 DParmsNVDQ. This table contains the fixed effects from the mixed model of density (dependent variable) as a function of sex, group (LD versus TD), chronological age (centered at 18 months), NVDQ (centered at the sample mean), the NVDQ-by-group interaction, and the age-by-group interaction. A separate model was specified for each electrode and frequency combination.

Table #9 DParmsVDQ. This table contains the fixed effects from the mixed model of density (dependent variable) as a function of sex, group (LD versus TD), chronological age (centered at 18 months), VDQ (centered at the sample mean), the VDQ-by-group interaction, and the age-by-group interaction. A separate model was specified for each electrode and frequency combination.

Table #10 DParmsSOC. This table contains the fixed effects from the mixed model of density (dependent variable) as a function of sex, group (LD versus TD), chronological age (centered at 18 months), Socialization (centered at the sample mean), the Socialization-by-group interaction, and the age-by-group interaction. A separate model was specified for each electrode and frequency combination.

Table #11 DSlopesNVDQ. This table contains the simple slopes of NVDQ within each group (LD and TD) from the mixed model of density (dependent variable) as a function of sex, group (LD versus TD), chronological age (centered at 18 months), NVDQ (centered at the sample mean), the NVDQ-by-group interaction, and the age-by-group interaction. A separate model was specified for electrode and frequency combination.

Table #12 DSlopesVDQ. This table contains the simple slopes of VDQ within each group (LD and TD) from the mixed model of density (dependent variable) as a function of sex, group (LD versus TD), chronological age (centered at 18 months), VDQ (centered at the sample mean), the VDQ-by-group interaction, and the age-by-group interaction. A separate model was specified for electrode and frequency combination.

Table #13 DSlopesSOC. This table contains the simple slopes of Socialization within each group (LD and TD) from the mixed model of density (dependent variable) as a function of sex, group (LD versus TD), chronological age (centered at 18 months), Socialization (centered at the sample mean), the Socialization-by-group interaction, and the age-by-group interaction. A separate model was specified for electrode and frequency combination.
